# Phosphorus and nitrogen co-limitation of forest ground vegetation under elevated anthropogenic nitrogen deposition

**DOI:** 10.1007/s00442-017-3945-x

**Published:** 2017-09-07

**Authors:** Per-Ola Hedwall, Johan Bergh, Jörg Brunet

**Affiliations:** 1 Southern Swedish Forest Research Centre, Swedish University of Agricultural Sciences, Sundsvägen 3, 230 53 Alnarp, Sweden; 2 Department of Forestry and Wood Technology, Linnaeus University, 351 95 Växjö, Sweden

**Keywords:** Mosses, Forbs, Graminoids, Eutrophication, Picea abies

## Abstract

Plant growth in northern forest ecosystems is considered to be primarily nitrogen limited. Nitrogen deposition is predicted to change this towards co-limitation/limitation by other nutrients (e.g., phosphorus), although evidence of such stoichiometric effects is scarce. We utilized two forest fertilization experiments in southern Sweden to analyze single and combined effects of nitrogen and phosphorus on the productivity, composition, and diversity of the ground vegetation. Our results indicate that the productivity of forest ground vegetation in southern Sweden is co-limited by nitrogen and phosphorus. Additionally, the combined effect of nitrogen and phosphorus on the productivity was larger than when applied solely. No effects on species richness of any of these two nutrients were observed when applied separately, while applied in combination, they increased species richness and changed species composition, mainly by promoting more mesotrophic species. All these effects, however, occurred only for the vascular plants and not for bryophytes. The tree layer in a forest has a profound impact on the productivity and diversity of the ground vegetation by competing for both light and nutrients. This was confirmed in our study where a combination of nitrogen and high tree basal area reduced cover of the ground vegetation compared to all the other treatments where basal area was lower after stand thinning. During the past decades, nitrogen deposition may have further increased this competition from the trees for phosphorus and gradually reduced ground vegetation diversity. Phosphorus limitation induced by nitrogen deposition may, thus, contribute to ongoing changes in forest ground vegetation.

## Introduction

The productivity of cold-temperate and boreal forest ecosystems is under natural conditions largely limited by nitrogen availability as indicated by fertilization experiments (Tamm 1991; LeBauer and Treseder 2008; Bobbink et al. 2010). For several decades, large areas have, however, been exposed to elevated anthropogenic nitrogen deposition (Dentener et al. 2006). Chronic nitrogen deposition has decreased the extent of nitrogen limitation, or even induced nitrogen saturation, which occurs when nitrogen input is larger than the retention capacity of the system. Nitrogen saturation is indicated by the lack of additional primary production after nitrogen fertilization and leakage of nitrogen and base cations (Aber et al. 1989). Such a lack of productivity response to nitrogen fertilization has been reported from areas with high or intermediate deposition (Bobbink et al. 2010; Tian et al. 2016). However, long-term fertilization experiments in boreal forests have indicated that nitrogen saturation does not occur as long as nitrogen is supplemented by other nutrients, such as phosphorus, even though the accumulated nitrogen addition is higher than in areas with high deposition (Bergh et al. 1999, 2008; Brockley 2010). This indicates that primary production is co-limited by other nutrients than nitrogen (Fay et al. 2015) and that nitrogen limitation may be induced anew by the addition of phosphorus or other nutrients. Moving away from the traditional view that primary production only is limited by one nutrient at the time (i.e., Liebig´s law of the minimum), there is ample evidence of co-limitations today, i.e., that plant growth can be limited by more than one nutrient at a time point (Harpole et al. 2011; Fay et al. 2015). Harpole et al. (2011) defined co-limitation strictly as either simultaneous or independent. An independent co-limitation is indicated by a positive effect on primary production of two or more nutrients individually, while a simultaneous co-limitation would require a positive growth response only when these are applied in combination. Although such information is available from multiple types of ecosystems, studies of forest vegetation are still rare, with a majority coming from tropical ecosystems (Elser et al. 2007).

The ground vegetation plays an important role in northern forest ecosystems because of its influence on soil processes, nutrient cycling, litter decomposition, forest succession, and provision of ecosystem services such as berry production (Nilsson and Wardle 2005; Sardans and Peñuelas 2012; Gamfeldt et al. 2013; Lindo et al. 2013). Along with increases in primary production, nutrient addition also influences forest ground vegetation diversity and composition by changing interspecific competition patterns (Bobbink et al. 2010; Gilliam 2006). While generally increasing primary production (Ceulemans et al. 2013; Meunier et al. 2016), anthropogenic nitrogen deposition is also considered to reduce plant species richness (Gilliam 2006) and to induce limitation of other nutrients, e.g., phosphorus (Vitousek et al. 2010). The impact of nitrogen, both from deposition and fertilization on understory vegetation, is fairly well studied (Gilliam 2006; Bobbink et al. 2010; Hedwall and Brunet 2016). Our understanding of the role of phosphorus limitation in forest ecosystems in general, and concerning understory vegetation in particular, is, however, very limited (Falkengren-Grerup et al. 1994). Hence, the effects of phosphorus on ecosystem productivity have been identified as a fundamental future research area for our understanding of ecosystem functioning (Sutherland et al. 2013). In temperate grassland ecosystems, plant species richness is generally negatively related to soil phosphorus (Ceulemans et al. 2014). High phosphorus concentrations in grasslands are mainly a legacy of former fertilization as phosphorus persists much longer in soils than nitrogen. In forests, only post-agricultural stands may have elevated phosphorus concentrations, and experimental fertilization is mainly done to study interactions with other nutrients and light (Baeten et al. 2010).

In this study, we used two adjacent field experiments in southern Sweden where nitrogen and phosphorus, separate and in combination, had been added to large (stand-scale) forested experimental plots. The main objective of this study was to increase our knowledge about nutrient limitation in forested ecosystems exposed to anthropogenic nitrogen deposition. Specifically we aimed to test the hypothesis that the combined effects of nitrogen and phosphorus on primary productivity, plant diversity, and composition are larger than the effects of nitrogen and phosphorus separately.

## Materials and methods

We used two forest fertilization experiments in south-west Sweden (56°41′, 13°6′) which were located in the vicinity of each other (maximum distance 5 km) and experience a similar climate and nitrogen deposition. The mean yearly temperature in the study area is 6.4 °C (average 1961–1990, weather station Simlångsdalen), the coldest month being January (−2.4 °C) and the warmest July (15.4 °C), while the mean annual precipitation is 1057 mm (www.smhi.se/klimatdata/meteorologi/temperatur/dataserier-med-normalvarden-1.7354). Total annual nitrogen deposition between 2005 and 2014 was 10.6 kg ha^−1^ year^−1^ (www.smhi.se/klimatdata/miljo/atmosfarskemi), which is high in comparison to most northern forests (Dentener et al. 2006). The forest stands were dominated by Norway spruce (*Picea abies*) established by planting between 1974 and 1983. The soils are podzols originating from sandy moraines or post-glacial sediments over an acid granite bedrock (www.sgu.se).

There were four treatments in the first experiment (from here on called Experiment 1): a control treatment with no fertilization (C), a nitrogen fertilization treatment (N), a nitrogen plus phosphorus fertilization treatment (NP), and a nitrogen fertilization treatment with high basal area of trees (NHBA). The latter treatment was established by thinning the tree layer in all other plots. The four treatments were assigned to 24 plots (737–1000 m^2^) at two closely located sites (1.8 km distance) with 12 plots at each site. Randomization was constrained within sites resulting in a balanced design with three replicates of each treatment at each site. Ammonium-nitrate (20 g N m^−2^) and calcium-dihydrogenphosphate (20 g P m^−2^) were applied in summer 2011. In summer 2012, additional phosphate (20 g P m^−2^) was applied resulting in a total dose of 40 g P m^−2^.

There were two treatments in the second experiment (from here on called Experiment 2): a control treatment with no fertilization (C), and a phosphorus fertilization treatment (P). The two treatments were randomly assigned to 12 plots (976–1009 m^2^) at one site resulting in a balanced design with six replicates per treatment. Calcium-dihydrogenphosphate (20 g P m^−2^) was applied in summer 2011 and another 20 g P m^−2^ was applied in summer 2012 (total dose 40 g P m^−2^). The large doses of both nitrogen and phosphorus were applied to ensure that none of these nutrients were limiting primary production. In the case of phosphorus, a reason was also to compensate for soil fixation, so-called sink-driven phosphorus limitation (Vitousek et al. 2010). The phosphorus was applied at two occasions to avoid leakage to the ground water, while the N dose applied normally causes very little leakage (Nohrstedt 2001).

The ground vegetation was surveyed during late summer 2016 (five growing seasons after the first fertilization). The total cover of both vascular plants and bryophytes was estimated in 20 systematically distributed (with equal distance along two transects) 0.25 m^2^ (0.5 m × 0.5 m) quadrats in each experimental plot. These cover estimates were used as a non-destructive method of biomass estimation due to the close positive correlation with true biomass (Röttgermann et al. 2000). Each quadrat was divided into a mesh of 25 squares of (0.1 m × 0.1 m). The abundance of individual plant species was estimated by counting in how many squares each species was present.

Leaf biomass of the grass *Avenella flexuosa* and the feather moss *Pleurozium schreberi* was collected in connection to the vegetation inventory and dried, milled, and analyzed for total nitrogen and phosphorus content. These two species were chosen because their nitrogen dynamics and response to fertilization are fairly well studied (e.g., Nordin et al. 1998). While *A. flexuosa* commonly is responding with increased growth to enhanced nitrogen availability, it has been shown that *P. schreberi* decreases in abundance after fertilization. Additionally, *A. flexuosa* was one of the most dominant species among the vascular plants at all sites, while *P. schreberi* was one of few bryophytes regularly present at all sites. Soil samples were taken in early June 2017. Ten subsamples were taken at each experimental plot and aggregated at the plot level (*n* = 36). The soil samples were then analyzed (methods within brackets) for pH (H_2_O, SS-ISO 10390:20), total phosphorus (SS028311/ICP-OE) and nitrogen (Leco FP-428), and organic matter (KLK 1965:1 mod) at an accredited laboratory (Eurofins Sweden).

All statistical analyses were done separately for the two experiments in R version 3.2.2. (R Core Team 2015). The effects of treatments on the cover of vascular plants and bryophytes, and on the leaf concentrations of nitrogen and phosphorus, were modeled by Generalized Linear Models (GLM) with a gamma error distribution and log-link. Similarly, a Poisson GLM with log-link was used to model the treatment effect on species richness (total number of species) of vascular plants and bryophytes, while the abundance of *A. flexuosa* was modeled with a negative binomial GLM (log-link). Planned contrasts between the following treatments were used to check for treatment effects: N vs. NP, N vs. NHBA, NP vs. NHBA. These were performed by applying the multcomp package (Hothorn and Westfall 2008) and *P* values were corrected for multiple comparisons by the FDR approach (Benjamini and Hochberg 1995). All GLMs were evaluated by plotting the residuals against the predicted values.

Non-metric multidimensional scaling (NMS) was performed on the community presence/absence data. The NMS analyses were done for the bryophyte and vascular communities separately and the treatments were projected on the ordination to check for effects of fertilization on species composition. The NMS was done by applying the metaMDS function and the treatment projections by the envfit function, both parts of the vegan package (Oksanen et al. 2016). Both the metaMDS and enfit functions were run with Bray–Curtis distance and 999 permutations restricted within site (for Experiment 1). To analyze if there were fundamental differences between the two experiments concerning our response variables, t-tests were used to check for differences between the control treatments of the two experiments regarding the six variables analyzed in the GLMs described above. None of these tests showed any significant differences between the experiments (*P* = 0.436–0.982). Due to the similarities between the two experiments concerning climate, background deposition, vegetation, and soil, we found the design appropriate to test hypotheses of independent co-limitation (see above) despite that the N and P treatments are located at different sites, while indications of simultaneous co-limitations still need to be interpreted with care.

## Results

### Soil conditions and nutrient concentrations in plant tissues

**Fig. 1:**
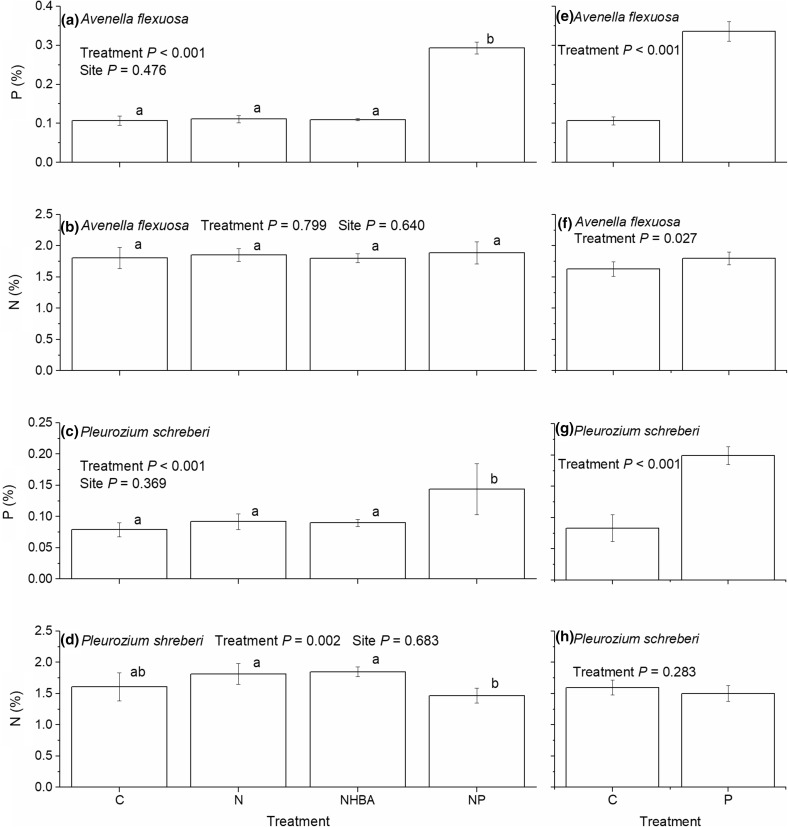
Phosphorus and nitrogen concentrations (mean ± 2SE) in leaves of *Avenella flexuosa* and *Pleurozium schreberi* for Experiment 1 (**a**–**d**) and Experiment 2 (**e**–**h**). Statistically significant differences (*P* < 0.05) between treatments are indicated by *different letters* above the *bars*. *C* control, *N* nitrogen fertilization, *NHBA* nitrogen fertilization and high basal area, *NP* nitrogen and phosphorus fertilization, *P* phosphorus fertilization

There were no effects of the treatments on pH, total content of nitrogen, or organic matter of the topsoil (0–10 cm), while both the NP and P treatments increased the content of phosphorous (Table 1). The N:P ratios in leaves of *A. flexuosa* and *P. schreberi* in control plots were 17.1 and 20.9 in Experiment 1, and 15.3 and 20.9 in Experiment 2, respectively (Table 2). Addition of phosphorus increased the concentrations of phosphorus in leaves of both species independently of whether phosphorus was added in combination with nitrogen or not (Fig. 1). The concentration increased approximately threefold in *A. flexuosa* (Fig. 1a, e) and doubled in *P. schreberi* (Fig. 1c, g) in comparison with the controls. Addition of solely nitrogen had no effects on leaf nitrogen concentrations in any of the two species (Fig. 1b, d, f, h). None of the nitrogen treatments had an effect on the phosphorus concentrations, but solely phosphorus resulted in 10% higher concentration of nitrogen in *A. flexuosa* than in the control (Fig. 1f). Additionally, nitrogen and phosphorus combined resulted in a 20% lower leaf nitrogen concentration in *P. schreberi* in comparison with the nitrogen treatments. Consequently, both the P and NP treatments decreased the N:P ratios of both species considerably (Table 2).

**Table 1:** Total nitrogen and phosphorus, organic matter, and pH (mean ± SE) of the 0–10 cm topsoil

Experiment	Treatment	pH (H_2_O)	Total N (mg kg^−1^)	Total P (mg kg^−1^)	Organic matter (%)
1 (*n* = 24)	C	4.28 ± 0.08	4353 ± 417	190 ± 14	19.4 ± 1.8
	N	4.27 ± 0.02	3080 ± 146	197 ± 9	16.8 ± 1.4
	NHBA	4.27 ± 0.04	3805 ± 273	203 ± 12	20.4 ± 1.7
	NP	4.35 ± 0.03	4465 ± 344	468 ± 22	16.6 ± 2.2
2 (*n* = 12)	C	4.27 ± 0.03	3630 ± 381	213 ± 19	12.6 ± 1.1
	P	4.25 ± 0.03	3123 ± 134	635 ± 42	14.1 ± 0.9

The first column of the table indicates from which of the two experiments, included in this study, the data originate

*C* control, *N* nitrogen fertilization, *NHBA* nitrogen fertilization and high basal area, *NP* nitrogen and phosphorus fertilization, *P* phosphorus fertilization

**Table 2:** Ratios of nitrogen and phosphorus (mean ± SE) of *Avenella flexuosa* and *Pleurozium schreberi* in the two experiments and treatments

Experiment	Treatment	*Avenella flexuosa*	*Pleurozium schreberi*
1 (*n* = 24)	C	17.05 ± 1.08	20.92 ± 2.04
	N	16.83 ± 0.84	19.90 ± 0.47
	NHBA	16.45 ± 0.33	20.69 ± 0.58
	NP	6.46 ± 0.33	13.41 ± 4.62
2 (*n* = 12)	C	15.32 ± 0.42	20.87 ± 3.05
	P	5.40 ± 0.25	7.56 ± 0.30

The first column of the table indicates from which of the two experiments, included in this study, the data originate

*C* control, *N* nitrogen fertilization, *NHBA* nitrogen fertilization and high basal area, *NP* nitrogen and phosphorus fertilization, *P* phosphorus fertilization

### Plant cover

**Fig. 2:**
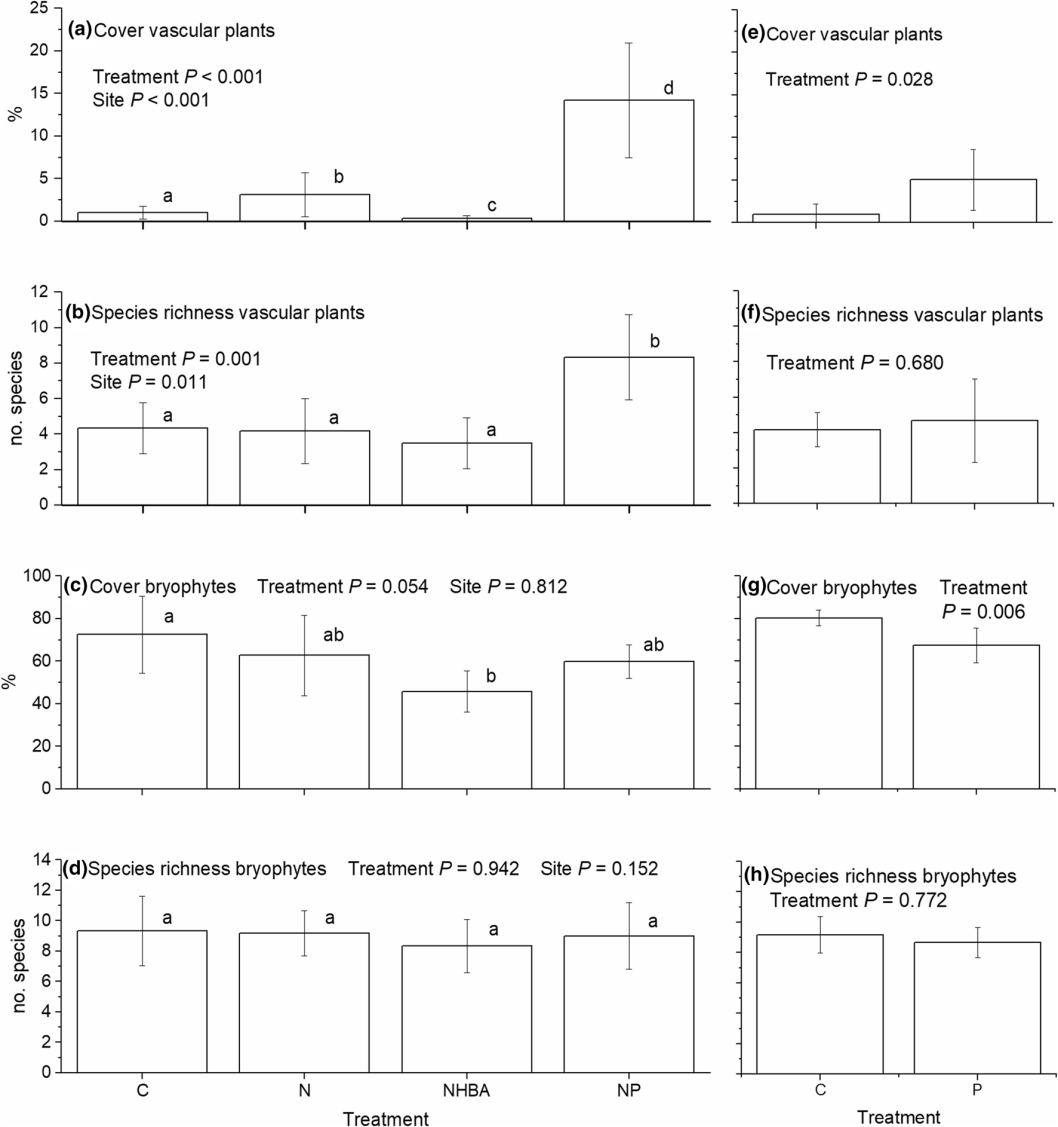
Total cover and total species richness (mean ± SE) for vascular plants and bryophytes for Experiment 1 (**a**–**d**) and Experiment 2 (**e**–**h**). Statistically significant differences (*P* < 0.05) between treatments are indicated by *different letters* above the *bars*. *C* control, *N* nitrogen fertilization, *NHBA* nitrogen fertilization and high basal area, *NP* nitrogen and phosphorus fertilization, *P* phosphorus fertilization

All treatments resulted in statistically significant effects on the total cover of vascular plants (Fig. 2a, e). Nitrogen addition alone caused three times higher cover than in the control treatment, while the cover in NHBA was only a third of the control (Fig. 2a). The largest cover increase of vascular plants was a result of the NP treatment, which had 14 times higher cover than in the control (Fig. 2a). Also phosphorus solely caused a higher cover (5 times) than in the control (Fig. 2e). The abundance of *A. flexuosa* increased as an effect of the NP and P treatments, while there were no effects of the other treatments (Table 3). The only effects found on the total cover of bryophytes were caused by the NHBA treatment and by the P treatment (Fig. 2c, g). Both had a lower cover compared with the controls.

**Table 3:** The mean abundance (hits m^−2^) and standard errors (SE) of *Avenella flexuosa* in the two experiments and treatments

Experiment	Treatment	Hits (m^−2^)	SE	*P* (site)	*P* (treatment)
1 (*n* = 24)	C	16.5 (a)	3.7	0.496	0.002
	N	21.2 (ab)	7.9		
	NHBA	9.6 (a)	3.6		
	NP	61.7 (b)	8.8		
2 (*n* = 12)	C	12.3	6.1		0.006
	P	41.6	9.9		

The *P* values come from Generalized Linear Models, and statistically significant differences (*P* < 0.05) between treatments are indicated by different letters within brackets. The first column of the table indicates from which of the two experiments, included in this study, the data originate

*C* control, *N* nitrogen fertilization, *NHBA* nitrogen fertilization and high basal area, *NP* nitrogen and phosphorus fertilization, *P* phosphorus fertilization

### Species richness and composition

**Fig. 3:**
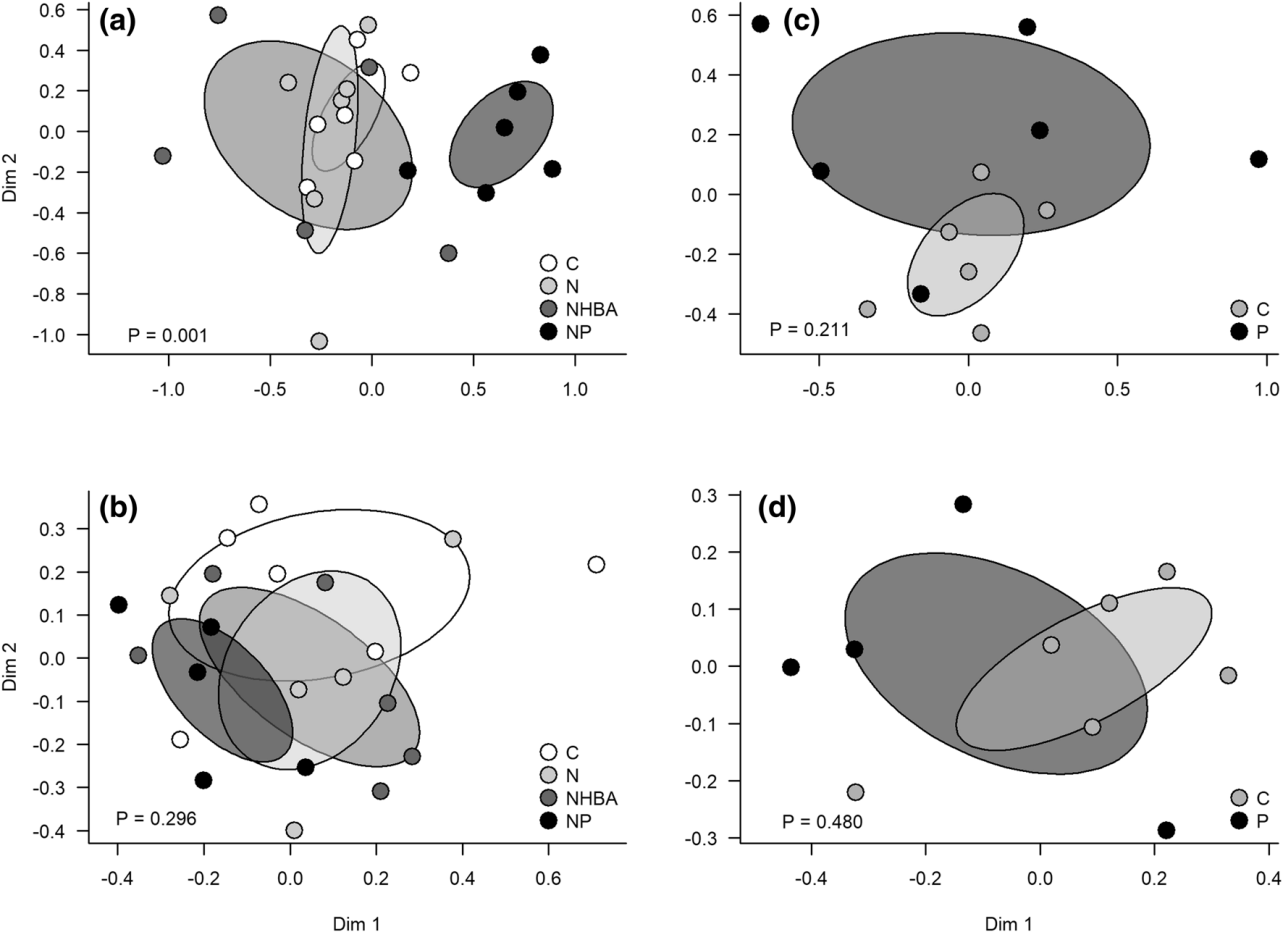
Ordination diagrams from non-metric multidimensional scaling for vascular plants (**a**, **c**) and bryophytes (**b**, **d**), respectively, in Experiment 1 (**a**, **b**) and 2 (**c**, **d**). *Ellipses* indicate 95% confidence intervals around the centroids of the treatments. *P* values indicate if there are effects of treatment on the location in ordination space. *C* control, *N* nitrogen fertilization, *NHBA* nitrogen fertilization and high basal area, *NP* nitrogen and phosphorus fertilization, *P* phosphorus fertilization

**Fig. 4:**
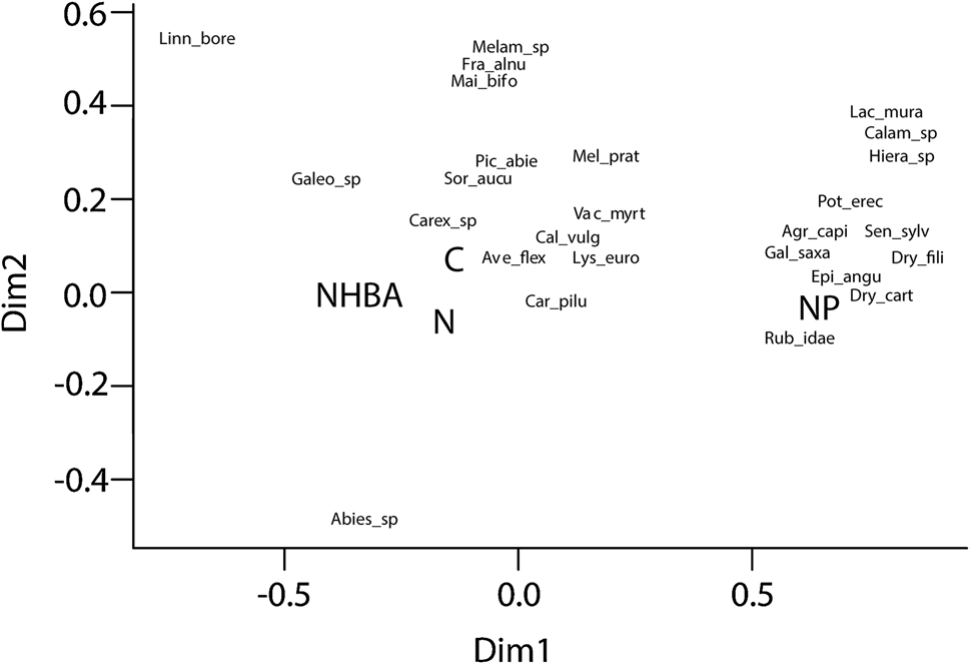
Ordination diagram from the non-metric multidimensional scaling for vascular plants in Experiment 1 where significant treatment effects were found (Fig. 3). *C,* control; *N,* nitrogen fertilization; *NHBA,* nitrogen fertilization and high basal area; *NP,* nitrogen and phosphorus fertilization; Abies_sp, *Abies* sp.; Ave_flex, *Avenella flexuosa*; Agr_capi, *Agrostis capillaris*; Calam_sp, *Calamagrostis* sp.; Cal_vulg, *Calluna vulgaris*; Car_pilu, *Carex pilulifera*; Carex_sp, *Carex* sp.; Dry_cart, *Dryopteris carthusiana*; Dry_fili, *Dryopteris filix*-*mas*; Epi_angu, *Epilobium angustifolium*; Fra_alnu, *Frangula alnus*; Galeo_sp, *Galeopsis* sp.; Gal_saxa, *Galium saxatile*; Hiera_sp, *Hieracium* sp.; Linn_bore, *Linnaea borealis*; Lys_euro, *Lysimachia europaea*; Mai_bifo, *Maianthemum bifolium*; Mel_prat, *Melampyrum pratense*; Melam_sp, *Melampyrum* sp.; Lac_mura, *Lactuca muralis*; Pic_abie, *Picea abies*; Pot_erec, *Potentilla erecta*; Rub_idae, *Rubus idaeus*; Sen_sylv, *Senecio sylvaticus*; Sor_aucu, *Sorbus aucuparia*; Vac_myrt, *Vaccinium myrtillus*

The only significant treatment effect on species richness was in the NP treatment. Addition of both nitrogen and phosphorus doubled the number of vascular plant species (Fig. 2b). Likewise, the only significant treatment effects on species composition were found among the vascular plants (Fig. 3a). As indicated by the 95% confidence intervals, it was only the NP treatment that deviated. There was also a tendency for an effect of solely phosphorus (Fig. 3c), but this was not statistically significant. The vascular species that characterized the NP treatment included both grasses, forbs and ferns (Fig. 4).

## Discussion

This study indicates that the productivity of forest ground vegetation in southern Sweden is simultaneously limited by nitrogen and phosphorus. While the phosphorus limitation most likely is aggravated by nitrogen deposition, nitrogen is still limiting growth despite the relatively high deposition that has prevailed for decades. Additionally, the combined effect of nitrogen and phosphorus on vascular plant abundance was larger than when both nutrients were applied separately, confirming our hypothesis. No effects on species richness of any of these two nutrients were observed when applied alone, while a combination of both increased the number of vascular plant species and changed overall species composition, again confirming our hypothesis. However, effects of the fertilization treatments on bryophyte vegetation were restricted to a slight decrease of total cover in the phosphorus treatment of Experiment 2.

The ratio between nitrogen and phosphorus in leaves has been suggested a predictor of whether plant communities are restricted by either or both of these nutrients (Tessier and Raynal 2003), and a ratio lower than 14 has been suggested to indicate nitrogen limitation and above 16 to indicate phosphorus limitation (Koerselman and Meuleman 1996). In between these limits the vegetation may be limited by any or both of these factors and the authors concluded that there is a considerable intraspecific variation. Accordingly, Güsewell (2004) later suggested a larger interval of co-limitation where ratios below 10 are to indicate nitrogen limitation and ratios above 20 to indicate phosphorus limitation (Güsewell 2004). This is in line with our results where the ratio of the clearly dominant vascular plant species, *A. flexuosa*, was between 15 and 17 indicating a somewhat larger limitation of phosphorus. Hence, both nutrients caused an increase in abundance, although the increase caused by phosphorus was much larger.

This pattern was, again, only visible among the vascular plants where phosphorus fertilization increased both biomass and species richness, as well as affected composition, but not so among the bryophytes despite an even higher ratio (20.9). Instead, phosphorus tended to decrease the cover of bryophytes, an effect likely to be due to increased competition from the vascular plants. In contrast to the vascular plants, the bryophyte cover was very high (73–80%), which may have restricted the potential response to fertilization of both biomass and species richness, as well as of the composition. The concentration of phosphorus in leaves of *P. schreberi* was significantly elevated in plots fertilized with this nutrient, while the concentration of nitrogen was lower when nitrogen was combined with phosphorus than when given alone. This may indicate dilution effects due to increased growth of *P. schreberi* in the NP treatment. However, no growth increase was indicated by the abundance data of this species (not shown), possibly because inefficient sampling as this species had a relatively low abundance in the experiments. Bryophytes have, relatively to vascular plants, low growth rates. Hence, the full fertilization effect on this species group may not have been fully realized during the rather short duration since the first fertilization (5 years). Fertilization with phosphorus slightly increased the concentration of nitrogen in leaves of *A. flexuosa* in Experiment 2. Addition of phosphorus has previously been shown to increase nitrogen availability in soils (e.g., Seastedt and Vaccaro 2001) which may have been the case here.

The NP treatment led to a higher number of vascular plant species, an effect that was absent both in the N and P treatments, which would indicate a simultaneous co-limitation on species richness, also supported by the N:P ratios (see above). The increasing species were all species that are common in the Swedish forest and were both disturbance-dependent species (e.g., *Senecio sylvaticus*) and species that are less dependent on disturbance (e.g., *Potentilla erecta*, *Dryopteris* spp.; Tyler and Olsson 2013). Compared to the other treatments, the vascular plant species that characterized the NP treatment also contained more indicators of relatively nutrient-rich conditions, e.g., raspberry *Rubus idaeus* and the herbs *Lactuca muralis* and *Epilobium angustifolium* (Tyler and Olsson 2013). Species recruitment is limited by both dispersal and establishment success (Baeten et al. 2009). Since the treatments were located at different sites, we cannot fully rule out an influence of land-use history on, for example, the seed banks or landscape effects on plant colonization. All species found are, however, common in the surrounding landscape (Hedwall and Brunet 2016) and easily dispersed.

Güsewell and colleagues (2005) suggested that phosphorus fertilization can increase species richness in wetlands when the N:P ratio is above 20 (15–17 in our study), indicating clear phosphorus limitation. This suggestion, however, builds on the assumption of relatively symmetric competition, which may be present in grassland communities, but was not the case in our study with low density of understory vascular plants and a dense tree layer. Most empirical evidence of the impact of phosphorus on productivity, species richness, and composition of the ground vegetation comes from studies in grasslands or other open environments (Güsewell 2004; Ceulemans et al. 2013, 2014). Evidence from the few existing field experiments in northern forests shows that effects of phosphorus are species dependent. Phosphorus application in a beech forest slightly increased shoot growth in three of six forest herbs tested, while no effects were found on the sporophore production or species richness of ectomycorrhizal or decomposer macrofungi during the five-year study (Falkengren-Grerup et al. 1994). In a pot experiment under field conditions, phosphorus application increased the uptake in all four forest herb species tested, but only increased growth in two of them (Baeten et al. 2010). Hence, the effects of phosphorus fertilization may be strongly community dependent. However, Güsewell et al. (2005) found that fewer wetland plant species were adapted to low phosphorus availability than to low nitrogen availability. If this represents a general pattern, it may be a plausible explanation behind the increase in species richness observed in our study. This pattern has, however, been indicated to be invert looking at only endangered species (Wassen et al. 2005) or grassland species (Ceulemans et al. 2014), stressing the difficulties of extrapolating across plant communities.

The tree layer in a forest has a profound impact on the ground vegetation by strongly restricting the light availability in the understory, and by competing for nutrients and water. Commonly, the trees constitute a large share of the photosynthetic biomass, which makes them, together with their mycorrhizal symbionts, a considerable nutrient sink. This implies that the response of the ground vegetation to changes in nutrient availability may be just as dependent on competition from the trees and their response to fertilization or deposition as on competition within the ground vegetation strata (e.g., Hedwall et al. 2010). This is further supported by the absence of nitrogen effects on vascular plant cover in plots with high basal area. Here the response of the vegetation was clearly limited by restricted light availability, but probably also by competition for nutrients from the trees (Barbier et al. 2008), which is supported by the productivity increase induced by nitrogen that took place in the N treatment despite the large input from deposition.

The vascular plant vegetation in the stands used in this study was initially poorly developed due to strong competition from the trees. Although light availability clearly plays a role in this, the response to fertilization stresses that it is not only light, but also nutrients, that play a role in these between-strata interactions. The competition from the tree layer may be strongly asymmetric and the trees with their ectomycorrhizal associations are strong competitors for phosphorus (Smith and Read 2008). The strong sink-effect from the trees and soil, and the associated microbes, for phosphorus is illustrated by clearly elevated needle phosphorus concentrations even several years after fertilization (Bergh, unpublished data) and by a study on nutrient leakage from Bahr et al. (2015) in Experiment 1. Despite the high dose applied, no elevated amounts of phosphorus were found in ground water below the main root zone of the trees (50 cm) during the years following fertilization. Nitrogen deposition may have further increased this competition from the trees for phosphorus and reduced understory plant diversity beyond the effects of just a reduction in light availability. Phosphorus limitation induced by nitrogen deposition may, thus, be a mechanism behind the diversity loss in forests generally observed as an effect of increased nitrogen input (Gilliam 2016).
